# A258 ULTRASOUND ASSESSMENT OF CROHN’S DISEASE PATIENTS FOLLOWING RISANKIZUMAB INITIATION; BASELINE, 3 AND 6 MONTH FOLLOW UP

**DOI:** 10.1093/jcag/gwad061.258

**Published:** 2024-02-14

**Authors:** R E Rosentreter, R Ingram, C Ma, M Chan, T Shukla, S Devlin, G G Kaplan, C Seow, R Panaccione, K Novak, C Lu

**Affiliations:** Medicine, University of Calgary Cumming School of Medicine, Calgary, AB, Canada; Medicine, University of Calgary Cumming School of Medicine, Calgary, AB, Canada; Medicine, University of Calgary Cumming School of Medicine, Calgary, AB, Canada; Medicine, University of Calgary Cumming School of Medicine, Calgary, AB, Canada; Medicine, University of Calgary Cumming School of Medicine, Calgary, AB, Canada; Medicine, University of Calgary Cumming School of Medicine, Calgary, AB, Canada; Medicine, University of Calgary Cumming School of Medicine, Calgary, AB, Canada; Medicine, University of Calgary Cumming School of Medicine, Calgary, AB, Canada; Medicine, University of Calgary Cumming School of Medicine, Calgary, AB, Canada; Medicine, University of Calgary Cumming School of Medicine, Calgary, AB, Canada; Medicine, University of Calgary Cumming School of Medicine, Calgary, AB, Canada

## Abstract

**Background:**

Risankizumab (RIS), an interleukin-23 inhibitor specific to the p19 subunit, was approved for the treatment of moderate to severe Crohn’s Disease (CD) in December 2022. Intestinal ultrasound (IUS) is an accurate and well-tolerated modality for evaluating response to therapy.

**Aims:**

We aim to assess IUS response at 3 and 6 months following RIS therapy.

**Methods:**

Consecutive patients initiating RIS for CD were recruited to be evaluated by IUS at baseline (within 14 days of initiation), 3 months, and 6 months. Baseline demographics, medication exposure, surgical history, and inflammatory biomarkers were obtained. IUS response (decrease in bowel wall thickness (BWT) ≥ 25%)) and transmural remission (TR) (normalization of BWT normalization, color doppler signal (CDS, modified Limberg (ML) score 0), wall stratification, inflammatory fat) for each segment were evaluated. Paired t-tests compared values at baseline, 3 and 6 months.

**Results:**

Thirty-three (20, 60.6% female) patients were recruited; 30 completed baseline and 3 month IUS, while 18 patients have 6 month IUS. Median age was 55 years (IQR 39-63), with a 12 year median CD duration (IQR 5-28). Five had inflammatory, 19 stricturing, and 6 fistulizing behaviour. CD distribution was 18 ileal, 1 colonic, and 11 ileocolonic. 20% (6/30) were bio-naïve, while 30% (9/30) had failed ≥ 3 biologics. Baseline mean CRP (4.1 ± 4.9 mg/L) did not significantly differ at 3 months (5.0 ± 6.8 mg/L, p=0.16), nor at 6 months for patients with values available (6.2mg/L ± 8.3, p = 0.38). Of patients with FC concentrations, 3 month mean FC (129.4 ±70 mcg/g) (9 patients) had significantly decreased from baseline (427.6 ± 271 mcg/g) (p=0.008), and similarly at 6 months (9 patients; baseline 396 ± 299 vs. 129 ± 75, p = 0.02). 80.0% (4/5) patients with colonic BWT ampersand:003E 3mm had IUS response (baseline mean BWT 5.6 ±1.4mm vs 3.5 ± 0.8mm at 3 months (p=0.011). IUS response was not achieved in those with ileal CD at 3 months (BWT 6.3 ± 2.1mm vs 3 month 6.0 ± 2.5mm (p=0.31). However, 21.4% (3/14) of ileal CD patients had an IUS response at 6 months. There is a non-significant ileal BWT reduction (5.4 ± 1.6mm) from baseline (6.0 ± 1.7mm) at 6 months. Of the 9 with moderate to severe CDS, 55% (5/9) had reduction to mild CDS by 3 months. TR was achieved by one patient with ileal CD at 6 months.

**Conclusions:**

IUS response was achieved in 80.0% of CD patients with colonic disease at 3 months follow RIS initiation. IUS response was not detected for ileal CD at 3 months, but detected in 21.4% at 6 months A significant FC reduction by 3 and 6 months was attained. Further prospective patient recruitment and IUS at 6 and 12 months to assess preliminary results and longer-term response is needed.

**Funding Agencies:**

None

